# Transpiration and Viscous Dissipation Effects on Entropy Generation in Hybrid Nanofluid Flow over a Nonlinear Radially Stretching Disk

**DOI:** 10.3390/e20090668

**Published:** 2018-09-04

**Authors:** Umer Farooq, Muhammad Idrees Afridi, Muhammad Qasim, D. C. Lu

**Affiliations:** 1 Department of Mathematics, Faculty of Science, Jiangsu University, Zhenjiang 212013, China; 2 Department of Mathematics, COMSATS University Islamabad, Park Road, Tarlai Kalan, Islamabad 455000, Pakistan

**Keywords:** hybrid nanofluid, entropy generation, Bejan number, nonlinear stretching, porous disk, viscous dissipation

## Abstract

The present research work explores the effects of suction/injection and viscous dissipation on entropy generation in the boundary layer flow of a hybrid nanofluid (Cu–Al_2_O_3_–H_2_O) over a nonlinear radially stretching porous disk. The energy dissipation function is added in the energy equation in order to incorporate the effects of viscous dissipation. The Tiwari and Das model is used in this work. The flow, heat transfer, and entropy generation analysis have been performed using a modified form of the Maxwell Garnett (MG) and Brinkman nanofluid model for effective thermal conductivity and dynamic viscosity, respectively. Suitable transformations are utilized to obtain a set of self-similar ordinary differential equations. Numerical solutions are obtained using shooting and bvp4c Matlab solver. The comparison of solutions shows excellent agreement. To examine the effects of principal flow parameters like suction/injection, the Eckert number, and solid volume fraction, different graphs are plotted and discussed. It is concluded that entropy generation inside the boundary layer of a hybrid nanofluid is high compared to a convectional nanofluid.

## 1. Introduction

The performance of an engineering systems gets degraded due to the presence of irreversibilities such as friction, mixing of fluids, non-quasi-static compression or expansion, chemical reactions, heat flow because of a temperature gradient, and unrestrained expansion (such as explosion). These irreversibilities bring an increase in system entropy and entropy created by such effects during any thermal process; this is called entropy generation. Entropy generation is used to establish the criteria for determining the performance of engineering devices. During the last few decades researchers and scientists in the field have re-examined energy conversion devices, identified the factors which cause the loss of quality of energy, and proposed different tools and methods to minimize the entropy. Bejan [1] for the first time introduced the idea of entropy generation minimization (EGM) in order to improve the thermal efficiency of engineering systems. After the remarkable work of Bejan many researchers and scientists performed the second law analysis under different flow and thermal conditions. Recently, Reddy et al. [2] studies the effects of a couple of stresses and magnetic fields on entropy generation in an unsteady flow of nanofluids over a vertical flat plate. Afridi and Qasim [3] examined the nonlinear Rosseland thermal radiation effects on entropy generation in the dissipative flow over a thin needle. Second law analysis of three-dimensional flow in the presence of frictional heating has been performed by Afridi and Qasim [4]. Khan et al. [5] investigated the entropy generation in an unsteady boundary layer flow over a vertical plate under the ramped wall temperature. Rashad et al. [6] analyzed the entropy generation rate in the nanofluid flow inside a square cavity in the presence of a heat sink/source at different locations with different sizes. Qasim and Afridi [7] discussed the entropy generation in the mixed convection flow of viscous fluid with temperature dependent thermal conductivity. The influence of ramped wall temperature and a porous medium on entropy generation in an electrically conducting fluid flowing over a vertical surface is reported by Khan et al. [8]. Afridi and Qasim [9] reported the comparative analysis of entropy generation in Cu–water and Ag–water nanofluids flowing over an elastic stretching sheet of variable thickness. Butt and Ali [10] numerically analyzed the effects of the Lorentz force on heat transfer and entropy generation in an unsteady boundary layer flow. The second law analysis of hydromagnetic flow over a radially stretching surface with Newtonian heating is performed by Das et al. [11]. The effects of diffusion-thermo and thermo-diffusion on entropy generation in a MHD rotating flow of Power–Eyring fluid are examined by Khan et al. [12]. A study of heat transfer and entropy analysis of a rotating flow of micropolar fluid was done by Khan et al. [13].

The viscous dissipation (frictional heating) is a conversion of kinetic energy of fluid to thermal energy due to the work done against the viscous forces. The dissipation of energy due to frictional heating (viscous heating) has significant importance in the boundary layer flows due to the high velocity gradients inside the boundary layer. Viscous dissipation is characterized by the Eckert number. For first time, Gebhart [14] studied the influence of viscous dissipation on the temperature in the natural convective flow. Partha et al. [15] reported the similarity solution of mixed convection flow in the presence of viscous dissipation. The effects of thermal radiation and viscous dissipation on temperature distribution in a boundary layer flow passing over a nonlinear stretching surface were studied by Cortell [16]. Recently, Sreenivasulu et al. [17] presented the effects of viscous dissipation, thermal radiation, and Joule heating on a MHD slip flow over a porous stretching surface. 

The word “nanofluids” was termed by Choi [18] which he used for the fluids with dispersed nanometer sized (1–100 nm) particles called nanoparticles. These nanoparticles are suspended in some base fluid (such as water, ethylene glycol, engine oil, etc.). Generally solid nanometer sized particles are of metals, (such as Al, Cu, or Ag) nonmetals (carbon nanotubes or graphite) oxides (Al_2_O_3_, TiO_2_, or CuO) carbides, nitrides, etc. Nanofluids have exceptional distinctive features like high thermal conductivity at lower nanoparticle aggregation, minimal clogging in flow passages, homogeneity, and long-term stability. Such distinctive features make them more useful in a variety of areas, such as in electronic applications (cooling of microchips, fluidic digital display devices, micro-electromechanical systems, microreactors, etc.), microfluidics, heating and cooling of buildings, lubricant systems, heat interchangers, refrigeration of electronic apparatus, pharmaceutical processes, transportation industry, in the biomedical field (nanodrug delivery, cancer therapeutics, cryopreservation, nano cryosurgery, sensing, and imaging etc.), and many others. Due to the large industrial and engineering applications of nanofluids, Makinde [19] investigated the viscous dissipation effects on boundary layer flow of nanofluids by taking metallic and nonmetallic nanoparticles. Hussanan et al. [20] studied the unsteady free convection flow of micropolar nanofluids over a vertical plate. The magnetohydrodynamic flow of ferrofluid over a thin needle has been investigated by Sulochana et al. [21]. Reddy and Chamkha [22] studied the flow of nanofluid over a horizontal circular cylinder saturated in a porous medium. The influence of a magnetic field on ferrofluid flow over a stretching/shrinking sheet was studied by Hussanan et al. [23]. Mutuku and Makinde [24] examined the effects of double stratification on the nanofluid flow over a flat surface. Some of the recent studies on viscous fluid and convectional nanofluid are presented in past papers [25,26,27,28,29,30,31,32].

The next step in nanofluids technology is the introduction of hybrid nanofluids, which are basically a dilute suspension of two or more types of nanoparticles in mixture or composite form. Hybrid nanofluids are prepared to overcome the disadvantages of individual suspension and harness the synergetic effect of nanoparticles. Hybrid nanofluids characterized the improved heat transfer and thermal conductivity of nanofluids leading to cost reduction in industrial applications. Experimental, theoretical, and numerical studies on hybrid nanofluids are very limited. Devi and Devi [33] examined the magnetic effects on hybrid nanofluids over a porous stretching surface in the absences of viscous dissipation. Chamkha et al. [34] numerically studied the water based hybrid nanofluid flow in a semicircular cavity by neglecting frictional heating. Devi and Devi [35] investigated the influence of the Lorentz force on three-dimensional flows of hybrid nanofluids. An entropy generation analysis of a hybrid nanofluid flow over a flat plate with convective boundary condition was performed by Olatundun and Makinde [36].

In the present work, our aim is to investigate the entropy generation and heat transfer analysis of boundary layer flow over a nonlinear radially stretching porous disk in the presence of viscous dissipation. The reduced governing equations are self-similar in nature and solved numerically using the Matlab in-built bvp4c solver and shooting technique. The obtained numerical solutions are utilized to compute entropy generation and Bejan number. Different graphs are plotted and discussed physically in order to explore the effects of various embedding flow control parameters.

## 2. The Mathematical Model

**Figure 1:**
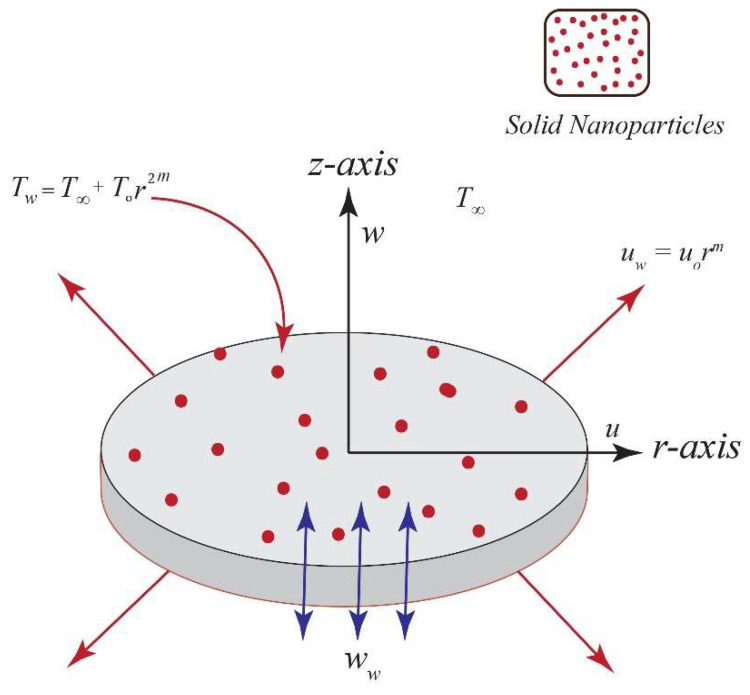
The geometrical representation of the flow problem over a radially stretching disk.

We consider the steady two-dimensional flow of an incompressible Cu–Al_2_O_3_–H_2_O hybrid nanofluid flow over a nonlinear radially stretching disk. The disk is assumed to be porous and located in the plane *z* = 0. The flow geometry along with the coordinate system is shown in Figure 1.

Under the above assumptions and Prandtl boundary layer approximations, the mass, linear momentum, and energy conservation equations take the following form [11]

(1) $$\frac{\partial }{\partial r}\left(ru\right)+\frac{\partial }{\partial z}\left(rw\right)=0,$$

(2) $$\rho_{hnf}\left(u\frac{\partial u}{\partial r}+w\frac{\partial u}{\partial z}\right)=\mu_{hnf}\left(\frac{\partial^{2}u}{\partial z^{2}}\right),$$

(3) $${\left(\rho c_{p}\right)}_{hnf}\left(u\frac{\partial T}{\partial r}+w\frac{\partial T}{\partial z}\right)=k_{hnf}\left(\frac{\partial^{2}T}{\partial z^{2}}\right)+\mu_{hnf}{\left(\frac{\partial u}{\partial z}\right)}^{2}.$$

The imposed boundary conditions are

(4) $$u(r,\;0)=u_{w}(r)=u_{o}r^{m},\;w(r,\;0)=w_{w}(r)=w_{o}r^{\frac{m-1}{2}},\;T(r,\;0)=T_{w}(r)=T_{\infty }+T_{o}r^{2m},$$

(5) $$u\to 0,\;T\to T_{\infty }\;\mathrm{as}\;z\to \infty ,$$

where $k_{hnf},$ $\mu_{hnf},$ ${\left(c_{p}\right)}_{hnf}$ and $\rho_{hnf}$ symbolize the thermal conductivity, dynamic viscosity, specific heat at constant pressure, and density of hybrid nanofluid, respectively.

### 2.1. Thermophysical Properties of Hybrid Nanofluid

This section presents the thermophysical properties of convectional nanofluid and hybrid nanofluid Cu–Al_2_O_3_–H_2_O.

#### 2.1.1. Effective Density

The effective density of the convectional nanofluid is denoted by $\rho_{nf}$ and given by [9]

(6) $$\rho_{nf}=\left(1-\phi \right)\rho_{bf}+\phi \rho_{s},$$

where, ϕ shows solid volume fraction, $\rho_{bf}$ represent density of base fluid, and $\rho_{s}$ indicates density of solid nanoparticles. So, the effective density $\left(\rho_{hnf}\right)$ of hybrid nanofluid is given by [34]

(7) $$\rho_{hnf}=\phi_{Al_{2}O_{3}}\rho_{Al_{2}O_{3}}+\phi_{Cu}\rho_{Cu}+\left(1-\phi_{Al_{2}O_{3}}-\phi_{Cu}\right)\rho_{bf},$$

where, $\rho_{hnf}$ represents density of hybrid nanofluid, $\rho_{Al_{2}O_{3}}$, $\rho_{Cu},$ $\phi_{Al_{2}O_{3}}$ and $\phi_{Cu}$ indicate density of $Al_{2}O_{3}$ solid nanoparticle, density of Cu solid nanoparticle, solid volume fraction of $Al_{2}O_{3}$ and Cu solid nanoparticle, respectively.

#### 2.1.2. Effective Heat Capacitance

The effective heat capacitance of regular nanofluid is given by [9]

(8) $${\left(\rho c_{p}\right)}_{nf}=\varphi {\left(\rho c_{p}\right)}_{s}+\left(1-\phi \right){\left(\rho c_{p}\right)}_{bf}.$$

The expression for effective heat capacitance of hybrid nanofluid ${\left(\rho c_{p}\right)}_{hnf}$ takes the following form [34]

(9) $${\left(\rho c_{p}\right)}_{hnf}=\left(1-\phi_{Al_{2}O_{3}}-\phi_{Cu}\right){\left(\rho c_{p}\right)}_{bf}+\phi_{Al_{2}O_{3}}{\left(\rho c_{p}\right)}_{Al_{2}O_{3}}+\phi_{Cu}{\left(\rho c_{p}\right)}_{Cu}.$$

#### 2.1.3. Effective Thermal Conductivity

According to Maxwell–Garnetts model [9], the thermal conductivity of regular nanofluid $k_{nf}$ is

(10) $$k_{nf}=\frac{\left(k_{s}+2k_{bf}\right)-2\phi \left(k_{bf}-k_{s}\right)}{\left(k_{s}+2k_{bf}\right)+\phi \left(k_{bf}-k_{s}\right)}k_{bf}.$$

So, the thermal conductivity of hybrid nanofluid containing spherical nanoparticles [34] is defined as

(11) $$\frac{k_{hnf}}{k_{bf}}=\frac{\left(\frac{\phi_{Al_{2}O_{3}}k_{Al_{2}O_{3}}+\phi_{Cu}k_{Cu}}{\phi_{Al_{2}O_{3}}+\phi_{Cu}}+2k_{bf}+2\left(\phi_{Al_{2}O_{3}}k_{Al_{2}O_{3}}+\phi_{Cu}k_{Cu}\right)-2\left(\phi_{Al_{2}O_{3}}+\phi_{Cu}\right)k_{bf}\right)}{\left(\frac{\phi_{Al_{2}O_{3}}k_{Al_{2}O_{3}}+\phi_{Cu}k_{Cu}}{\phi_{Al_{2}O_{3}}+\phi_{Cu}}+2k_{bf}-\left(\phi_{Al_{2}O_{3}}k_{Al_{2}O_{3}}+\phi_{Cu}k_{Cu}\right)-\left(\phi_{Al_{2}O_{3}}+\phi_{Cu}\right)k_{bf}\right)}$$

#### 2.1.4. Effective Dynamic Viscosity

The effective dynamic viscosity of the regular nanofluid $\mu_{nf}$ and hybrid nanofluid $\mu_{hnf}$ based on the Brinkman model [9,34] are respectively given by

(12) $$\mu_{nf}=\frac{\mu_{bf}}{{\left(1-\phi \right)}^{2.5}},$$

(13) $$\mu_{hnf}=\frac{\mu_{bf}}{{\left(1-\phi_{Al_{2}O_{3}}-\phi_{Cu}\right)}^{2.5}}.$$

### 2.2. Similarity Transformations

Employing the similarity transformations

(14) $$\xi =z\sqrt{\frac{u_{o}(m+1)}{2\nu_{bf}}}r^{\frac{m-1}{2}}z,\;u=u_{o}r^{m}g^{\prime }\left(\xi \right),\;w=u_{o}r^{\frac{m-1}{2}}\sqrt{\frac{2\nu_{bf}}{u_{o}(m+1)}}\left[\frac{m+3}{2}g\left(\xi \right)+\frac{m-1}{2}\xi g^{\prime }\left(\xi \right)\right],$$

(15) $$\;T=\theta \left(T_{w}-T_{\infty }\;\right)+T_{\infty }.$$

Equations (2) and (3) and the boundary conditions (4) and (5) take the following form

(16) $$\frac{g^{\prime \prime \prime }}{{\left(1-\phi_{Al_{2}O_{3}}-\phi_{Cu}\right)}^{2.5}\left(1-\phi_{Al_{2}O_{3}}-\phi_{Cu}+\frac{\rho_{Al_{2}O_{3}}\phi_{Al_{2}O_{3}}+\rho_{Cu}\phi_{Cu}}{\rho_{bf}}\right)}+\frac{m+3}{m+1}gg^{\prime \prime }-2\frac{m}{m+1}{g^{\prime }}^{2}=0,$$

(17) $$\frac{0.5\;}{\mathrm{Pr}}\frac{H_{1}}{H_{2}}\theta^{\prime \prime }+\frac{m+3}{2\left(m+1\right)}g\theta^{\prime }-\frac{2mg^{\prime }\theta }{m+1}+\frac{Ec{g^{\prime \prime }}^{2}}{2H_{2}{\left(1-\phi_{Al_{2}O_{3}}-\phi_{Cu}\right)}^{2.5}}=0,$$

(18) $$g\left(0\right)=g_{w},\;g^{\prime }(0)=1,\;\theta \left(0\right)=1,$$

(19) $$g^{\prime }\left(\xi \to \infty \;\right)=0,\;\theta \left(\xi \to \infty \right)=0.$$

where, $Ec=\frac{u_{w}^{2}}{{\left(c_{p}\right)}_{bf}\;\left(T_{w}-T_{\infty }\right)}=\frac{u_{o}^{2}}{{\left(c_{p}\right)}_{bf}T_{o}}$ (Eckert number), $H_{1}=\frac{k_{hnf}}{k_{bf}}$, $g_{w}=-\frac{w_{o}}{m+3}\sqrt{\frac{2\left(m+1\right)}{u_{o}\nu_{bf}}}$ (mass transfer parameter, positive for suction and negative for injection, $H_{2}=\left(1-\phi_{Al_{2}O_{3}}-\phi_{Cu}+\frac{{\left(\phi \rho c_{p}\right)}_{Al_{2}O_{3}}+{\left(\phi \rho c_{p}\right)}_{Al_{2}O_{3}}}{{\left(\rho c_{p}\right)}_{bf}}\right)$ and $\mathrm{Pr}=\frac{\nu_{bf}}{\alpha }$ (Prandtl number), α indicates fluid thermal diffusivity.

### 2.3. Entropy Generation

The rate of volumetric entropy generation due to the viscous irreversibility and heat transfer irreversibility in a two-dimensional boundary layer flow of hybrid nanofluid fluid is given by

(20) $${{\dot{S}}^{\prime \prime \prime }}_{Gen}=\frac{k_{hnf}}{T^{2}}{\left(\frac{\partial T}{\partial z}\right)}^{2}+\frac{\mu_{hnf}}{T}{\left(\frac{\partial u}{\partial z}\right)}^{2}.$$

The term $\frac{k_{hnf}}{T^{2}}{\left(\frac{\partial T}{\partial z}\right)}^{2}$ indicates the entropy generation due to heat transfer across the boundary layer, whereas the term $\frac{\mu_{hnf}}{T}{\left(\frac{\partial u}{\partial z}\right)}^{2}$, represents the entropy generation due to viscous dissipation. The characteristic entropy generation ${\left({{\dot{S}}^{\prime \prime \prime }}_{Gen}\right)}_{o}$ under the imposed boundary conditions has form:

(21) $${\left({{\dot{S}}^{\prime \prime \prime }}_{Gen}\right)}_{o}=\frac{k_{bf}u_{w}}{2r\nu_{bf}}.$$

Using Equation (21) and similarity transformations, we obtained the following dimensionless form of entropy generation

(22) $$Ns=\frac{{{\dot{S}}^{\prime \prime \prime }}_{Gen}}{{\left({{\dot{S}}^{\prime \prime \prime }}_{Gen}\right)}_{o}}=\left[\left(\frac{k_{hnf}}{k_{bf}}\right)\frac{{\theta^{\prime }}^{2}}{{\left(\theta +\Lambda \right)}^{2}}+\frac{Ec\mathrm{Pr}}{{\left(1-\phi_{Al_{2}O_{3}}-\phi_{Cu}\right)}^{2.5}\left(\theta +\Lambda \right)}{g^{\prime \prime }}^{2}\right]\left(m+1\right)$$

where $Ns=\frac{{{\dot{S}}^{\prime \prime \prime }}_{Gen}}{{\left({{\dot{S}}^{\prime \prime \prime }}_{Gen}\right)}_{o}}$ represents the dimensionless form of entropy generation and $\Lambda =\frac{T_{\infty }}{T_{w}-T_{\infty }}$ indicates the temperature difference parameter.

## 3. Numerical Results and Discussions

The second law and heat transfer analysis of Cu–Al_2_O_3_–water hybrid nanofluid and Al_2_O_3_–water regular nanofluid are performed in the presence of viscous dissipation and suction/injection effects. The dimensionless set of nonlinear differential equations are solved numerically by utilizing the Runge–Kutta–Fehlberg scheme. For validation of our numerical code, the set of nonlinear differential equations are also solved numerically by utilizing the Matlab in-built boundary value solver bvp4c. Our numerical results are found to be in an excellent agreement as shown in Table 1. The thermophysical properties of solid nanoparticles and base fluid (water) are displayed in Table 2.

**Figure 2:**
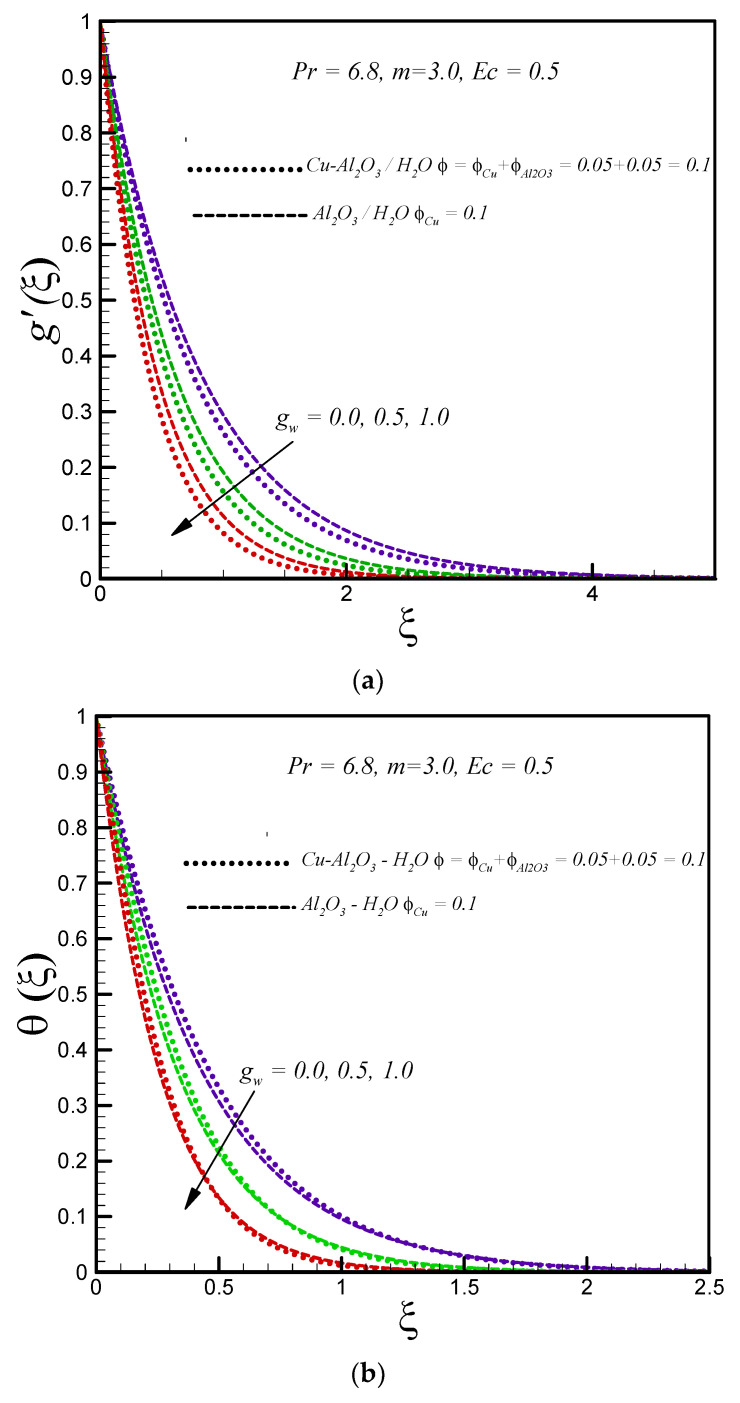

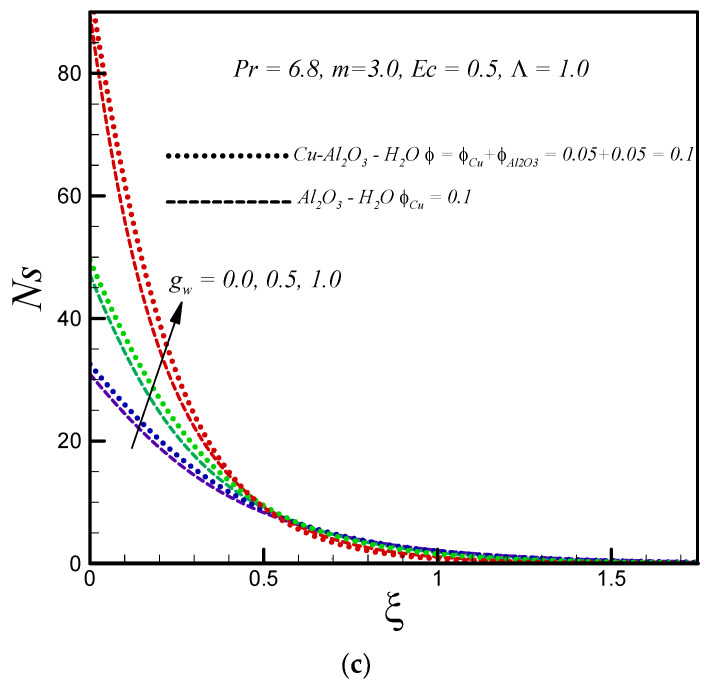
Effects of suction on (**a**) $g^{\prime }\left(\xi \right)$ (**b**) θ(ξ) (**c**) Ns.

Figure 2a explores the influence of mass suction parameter ($g_{w}>0$) on the velocity profile $g^{\prime }\left(\xi \right).$ It is noticed that the velocity and thickness of the velocity boundary layer decrease when enhancing the values of suction for both regular and hybrid nanofluids. Further, the velocity of the hybrid nanofluid is less (because of high viscosity) compared to the regular nanofluid. In addition, velocity reaches its maximum value in the case of using the impermeable disk for both type of nanofluids. The influence of suction on temperature distribution is shown in Figure 2b. Fluid suction ($g_{w}>0$) brings the ambient fluid near to the stretching disk and enhances the heat transfer. Thus, the thickness of the thermal boundary layer decreases with increasing mass suction. Figure 2c illustrates the influence of suction on the entropy generation number for both types of nanofluids. It is viewed that entropy generation increases at the surface and in vicinity of the disk, with enhancing values of mass suction. The minimum entropy generation is observed in case of the impermeable porous disk for both types of nanofluids. In addition, more entropy is generated in the boundary layer flow of the hybrid nanofluid.

**Figure 3:**
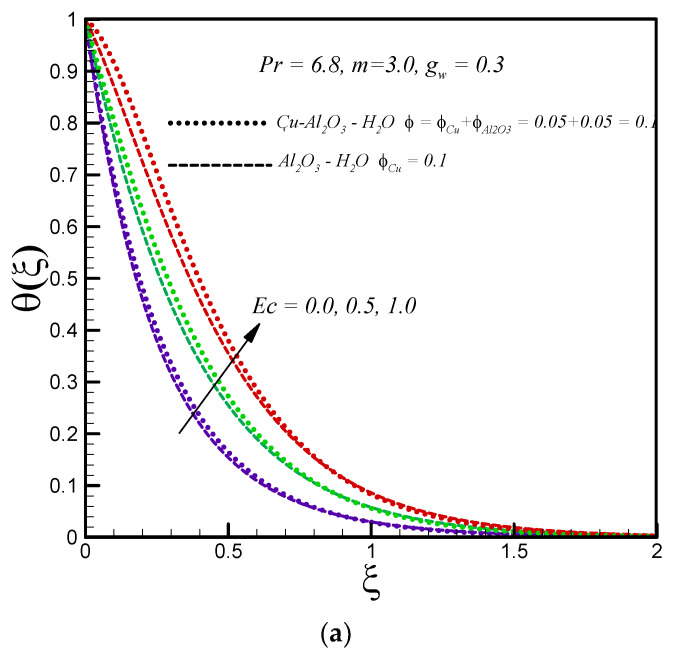

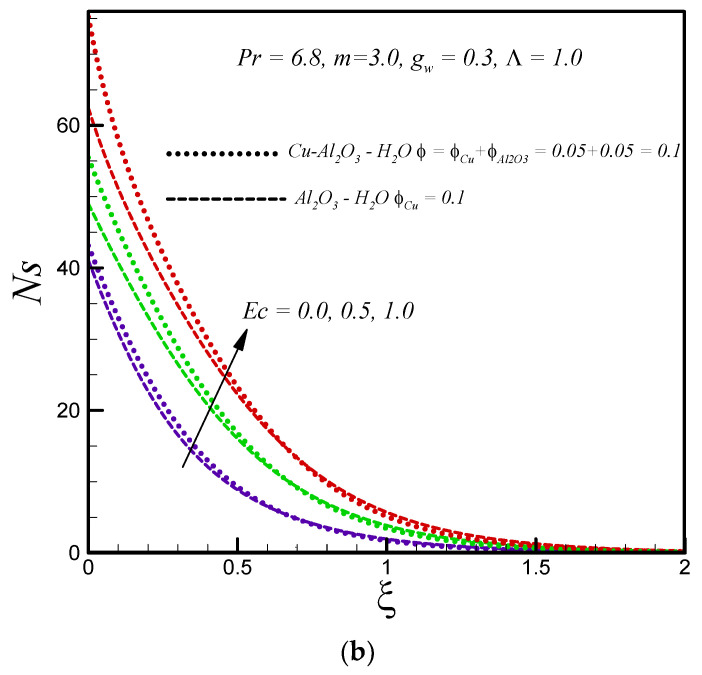
Effects of Ec on (**a**) θ(ξ) (**b**) Ns.

The variations of temperature profile of the hybrid nanofluid and convectional nanofluid with the viscous dissipation parameter (*Ec*) are depicted in Figure 3a. An increase in the *Ec* number leads to an increase in temperature of both types of nanofluids inside the boundary layer because of the friction between the adjacent layers of nanofluid. As a result, the kinetic energy is converted to internal energy and the conversion is characterized by an increase in temperature. The impact of the Eckert number on entropy generation is presented in Figure 3b. We noticed that entropy generation is an increasing function of *Ec* number; this is because viscous heating enhances with increasing *Ec* number, and as a result, the molecular disorderness increases and thereby leads to an increase in the entropy generation number. The increasing effects are more prominent at the surface of stretching disk for both types of nanofluids. Further, the hybrid nanofluid causes more entropy generation as compared to the convectional nanofluid. For fixed values of Ec number, entropy generation decreases for both types of nanofluids and asymptotically goes to zero as one moves towards the edge of the boundary layer.

**Figure 4:**
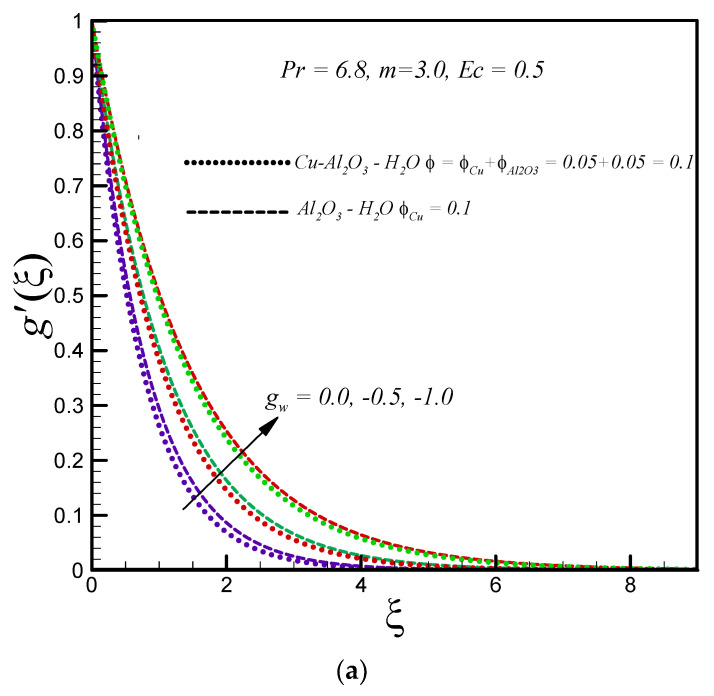

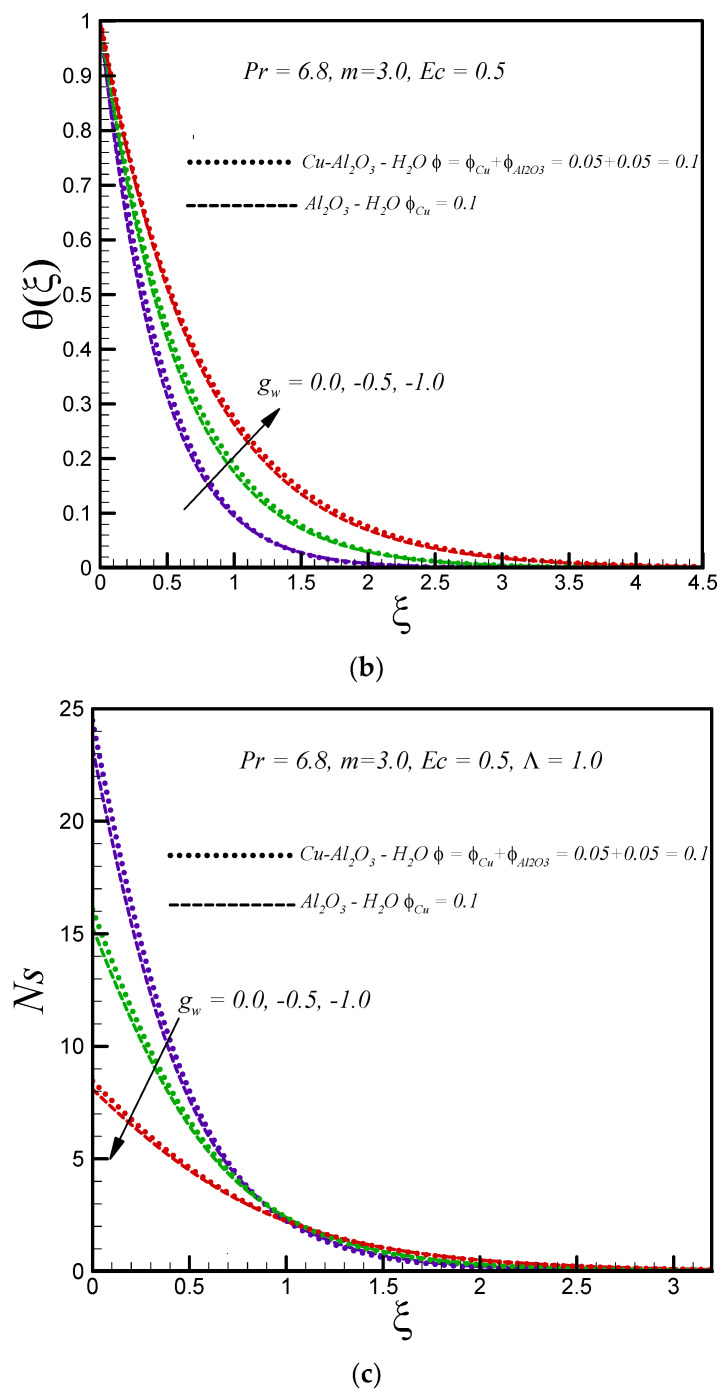
Effects of injection on (**a**) $g^{\prime }\left(\xi \right)$ (**b**) θ(ξ) (**c**) Ns.

Figure 4a depicts the effects of mass injection on the velocity field of the hybrid nanofluid (Cu–Al_2_O_3_–water) and the convectional nanofluid (Al_2_O_3_–water). It is found that the motion of both types of nanofluid is accelerated with rising values of mass injection. The increasing effect is higher in the convectional nanofluid compared to the hybrid nanofluid. In addition, the hydrodynamic boundary becomes thicker when the mass injection is applied. The temperature inside the boundary layer rises for both types of nanofluid with increasing mass injection, as shown in Figure 4b. Figure 4c demonstrates the influence of mass injection on the entropy generation number *Ns* for both types of nanofluids. It is noticed that *Ns* is a decreasing function of mass injection. An increase in mass injection parameters leads to a decrease in velocity and thermal gradients inside the boundary layer. As a result, the entropy generation number *Ns* decreases (because entropy generation is proportional to velocity and temperature gradients). Less entropy generation is observed for the regular nanofluid (Al_2_O_3_–water) compared to the hybrid nanofluid (Cu–Al_2_O_3_–water). Further, the decreasing effect of entropy generation is significantly more prominent at the surface of the stretching disk.

**Figure 5:**
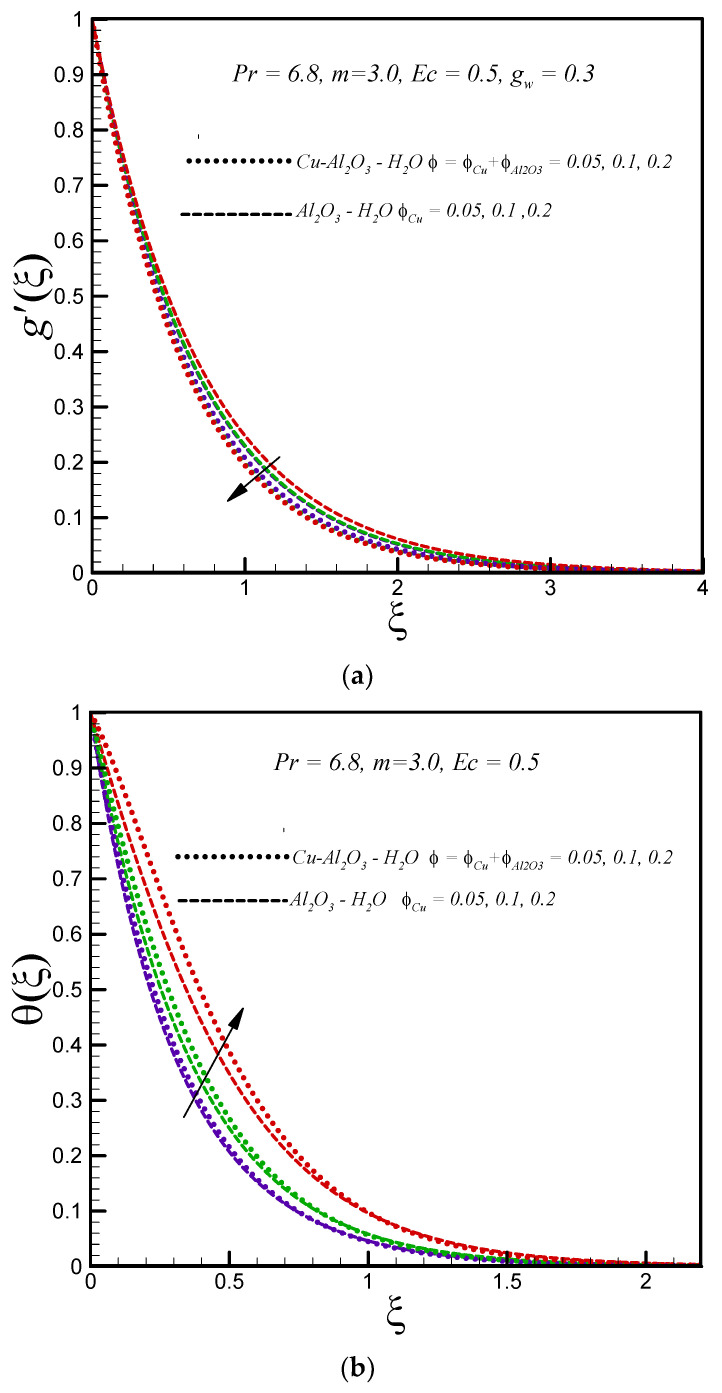

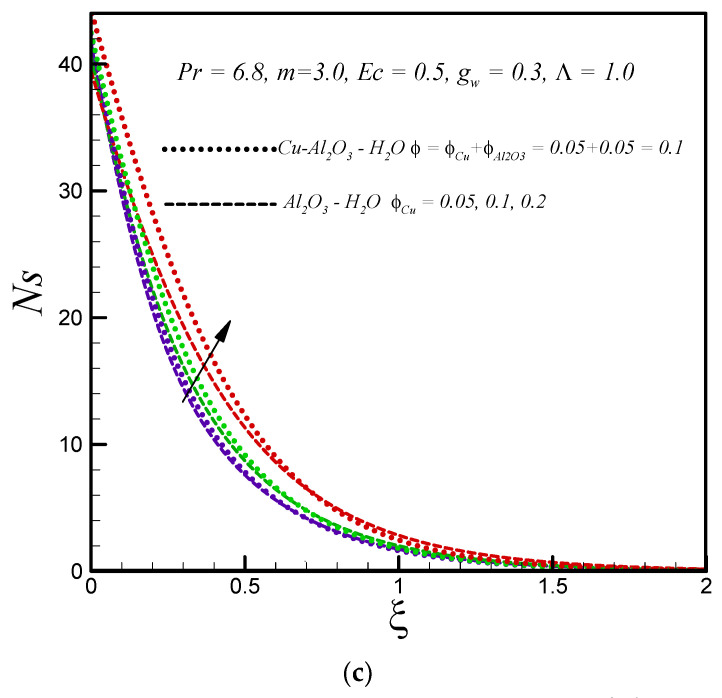
Effects of nanoparticles solid volume fraction on (**a**) $g^{\prime }\left(\xi \right)$ (**b**) θ(ξ) (**c**) Ns.

**Figure 6:**
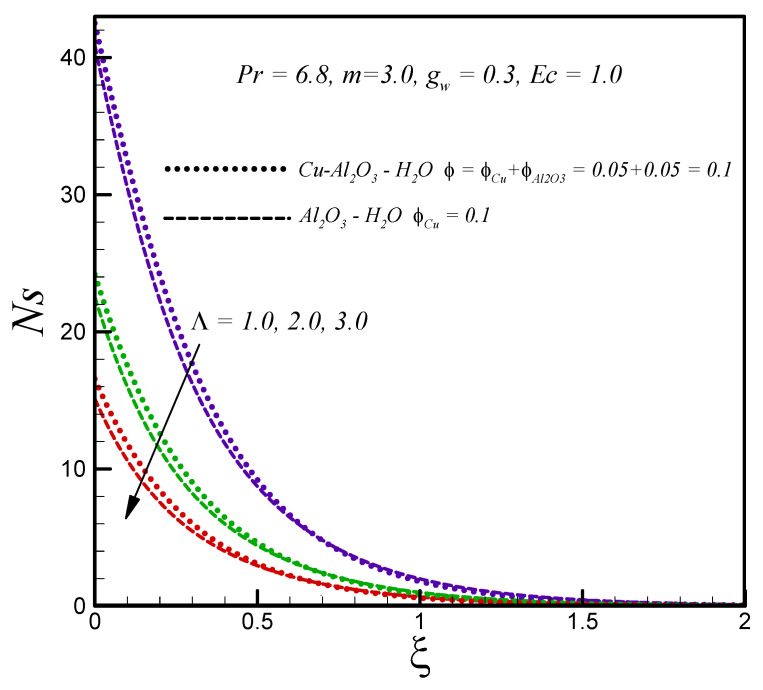
Effects of the temperature difference parameter on Ns.

Figure 5a shows the influence of the solid nanoparticle volume fraction on the velocity profile $g^{\prime }\left(\xi \right).$ The graphical results show that motion of both types of nanofluid decelerate with increasing nanoparticle volume fraction (due to increasing effective dynamic viscosity). In addition, it is found that for the hybrid nanofluid, the velocity is lower than the convectional nanofluid. The effects of the solid nanoparticle volume fraction on temperature distribution for both types of nanofluids is demonstrated in Figure 5b. The influence of the solid volume fraction is found to broaden the temperature distribution and thermal boundary layer thickness for both types of nanofluids. The thermal conductivity of nanofluids enhances with increasing nanoparticle volume fraction. Figure 5c aims to explore the influence of solid nanoparticle volume fraction on entropy generation. It is found that for both hybrid and regular nanofluids entropy generation increases with an increasing value of solid nanoparticle volume fraction. The reduction in entropy generation with an enhancing temperature difference parameter is observed as depicted in Figure 6.

The reduction in entropy generation is more prominent at the surface of the radially stretching disk for both types of nanofluids.

## 4. Concluding Remarks

The current study presents a numerical investigation of entropy generation inflow over a radially stretching disk influenced by viscous dissipation, suction/injection, and heat transfer in a conventional nanofluid Al_2_O_3_–H_2_O and hybrid nanofluid Cu–Al_2_O_3_–H_2_O. The main outcomes of the present study are summarized as follows:

- The velocity and temperature profile of regular and hybrid nanofluids decrease with increasing suction.
- The thermal boundary layer is thick for a hybrid nanofluid in comparison to a regular nanofluid.
- Increasing values of Eckert number, nanoparticle volume fraction, and injection result in a rise in temperature distribution for both the regular and hybrid nanofluid.
- Entropy generation increases with rising values of suction, Eckert number, and nanoparticle volume fraction for both types of nanofluid.
- Entropy generation in the hybrid nanofluid Cu–Al_2_O_3_–H_2_O is higher than regular nanofluid Al_2_O_3_–H_2_O.
- A reduction in entropy generation is observed with increasing temperature difference and injection.

## Figures and Tables

**Table 1:** Comparison of the numerical results of $-g^{\prime \prime }(0)$ and $-\theta^{\prime }\left(0\right)$ corresponding to the different values of $g_{w}>0$ when m=3.0, $\phi_{Al_{2}O_{3}}=0.1$ and $\phi_{Al_{2}O_{3}}+\phi_{Cu}=0.05+0.05=0.1$.

	Al_2_O_3_–Water				Cu–Al_2_O_3_–Water			
$g_{w}$	R-K-Fehlberg Scheme		bvp4c		R-K-Fehlberg Scheme		Bvp4c	
	$-g^{\prime \prime }(0)$	$-\theta^{\prime }(0)$	$-g^{\prime \prime }(0)$	$-\theta^{\prime }(0)$	$-g^{\prime \prime }(0)$	$-\theta^{\prime }(0)$	$-g^{\prime \prime }(0)$	$-\theta^{\prime }(0)$
0.1	−1.300363	−2.398908	−1.300361	−2.398909	−1.427521	−2.045604	−1.427521	−2.045602
0.3	−1.468136	−2.670735	−1.468135	−2.670737	−1.629400	−2.185050	−1.629401	−1.629400
0.5	−1.653273	−2.968903	−1.653271	−2.968903	−1.853650	−2.333798	−1.853651	−1.853652
0.7	−1.854387	−3.292556	−1.854387	−3.292555	−2.098183	−2.496151	−2.098182	−2.098181
0.9	−2.069710	−3.639854	−2.069711	−3.639854	−2.360413	−2.674312	−2.360413	−2.360412

**Table 2:** Thermophysical properties of base fluid and solid nanoparticles.

Physical Properties	Base Fluid (Water)	$Al_{2}O_{3}$	Cu
$c_{p}\;\left(J/kgK\right)$	4179	765	385
k (W/mK)	0.613	40	401
$\rho \;\left(kg/m^{3}\right)$	997.1	3970	8933
Pr	6.8	-	-
